# Detailed phenotyping reveals diverse and highly skewed neutrophil subsets in both the blood and airways during active tuberculosis infection

**DOI:** 10.3389/fimmu.2024.1422836

**Published:** 2024-06-14

**Authors:** Shepherd Nhamoyebonde, Mark Chambers, Lerato Ndlovu, Farina Karim, Matilda Mazibuko, Zoey Mhlane, Lindiwe Madziwa, Yunus Moosa, Sashen Moodley, Monjurul Hoque, Alasdair Leslie

**Affiliations:** ^1^ Africa Health Research Institute, Durban, South Africa; ^2^ Department of Infectious Diseases, Nelson R. Mandela School of Clinical Medicine, University of KwaZulu-Natal, Durban, South Africa; ^3^ Department of Infection and Immunity, University College London, London, United Kingdom

**Keywords:** neutrophil, tuberculosis, HIV, blood, sputum, PDL-1

## Abstract

**Introduction:**

Neutrophils play a complex and important role in the immunopathology of TB. Data suggest they are protective during early infection but become a main driver of immunopathology if infection progresses to active disease. Neutrophils are now recognized to exist in functionally diverse states, but little work has been done on how neutrophil states or subsets are skewed in TB disease.

**Methods:**

To address this, we carried out comprehensive phenotyping by flow cytometry of neutrophils in the blood and airways of individuals with active pulmonary TB with and without HIV co-infection recruited in Durban, South Africa.

**Results:**

Active TB was associated with a profound skewing of neutrophils in the blood toward phenotypes associated with activation and apoptosis, reduced phagocytosis, reverse transmigration, and immune regulation. This skewing was also apparently in airway neutrophils, particularly the regulatory subsets expressing PDL-1 and LOX-1. HIV co-infection did not impact neutrophil subsets in the blood but was associated with a phenotypic change in the airways and a reduction in key neutrophil functional proteins cathelicidin and arginase 1.

**Discussion:**

Active TB is associated with profound skewing of blood and airway neutrophils and suggests multiple mechanisms by which neutrophils may exacerbate the immunopathology of TB. These data indicate potential avenues for reducing neutrophil-mediated lung pathology at the point of diagnosis.

## Introduction

Tuberculosis (TB), caused by *Mycobacterium tuberculosis (M.tb)*, remains a severe global health challenge. Infection primarily occurs via the lung mucosa through inhalation of aerosol droplets containing *M.tb* (1). In the alveolar space *M.tb* encounters immune cells such as alveolar macrophages, pneumocytes, and lung endothelial cells, leading to the recruitment of other immune subsets such as neutrophils, eosinophils, and dendritic cells. Of these, neutrophils are the first cell type to be recruited in abundance from the circulation and are regarded as a first line of defense against invading pathogens (2–4).

The role of neutrophils in TB immunity is complex and is not fully understood. On one hand they play a role in bacterial clearance by promoting the transition from innate to adaptive immunity through the release of cytokines and chemokines (5–10). Additionally, neutrophils can drive granuloma formation via the CXCR3 pathway, a key regulator of inflammatory responses (11). Depletion of neutrophils following vaccination with *M. smegmatis* in mice, for example, resulted in decreased T-cell responses and increased *M.tb* burden (12). Studies have also demonstrated an inverse correlation between blood neutrophil counts and the risk of active TB in humans (13, 14). On the other hand, in *M.tb* susceptible mice, neutrophils have been observed driving severe lung inflammation (15, 16), and they are thought to be central to TB immunopathology and lung cavitation in humans.

Neutrophils were historically considered as a single immune subset, but more recent studies indicate the existence of heterogenous populations of neutrophils whose functional capacity varies (13, 17–19). *M.tb* infection affects the phenotype and function of circulating neutrophils (20), and the skewing of neutrophil phenotypes in TB might play an important role in disease progression (15). In addition, TB has been linked with the release of banded and immature neutrophils into circulation, which have differential phenotypic and functional characteristics (21). Likewise, HIV infection has been reported to decrease neutrophil chemotaxis, phagocytosis, and bactericidal activity, which may result in failure of the host to control *M.tb* infection (22–25). However, detailed studies examining the heterogeneity of neutrophils induced during active TB disease and the impact of HIV co-infection are missing, and we still lack a clear picture of how they might contribute to immune control or pathogenesis. In addition, the limited studies examining this important cell type have focused primarily on the blood, and it is not clear whether the same neutrophils populations are present at the site of infection in the human lung. The aim of this study was thus to perform an extensive functional immunophenotyping of blood and sputum neutrophils in TB-infected individuals, as a proxy of the lung environment.

These data show that neutrophils are highly skewed by TB infection, and display evidence of immune activation, functional impairment and immunosuppressive phenotypes. Surprisingly, we detected no significant effect of HIV-coinfection on neutrophil subsets in blood. Neutrophils were found to be highly enriched in the sputum of TB infected subjects, and were also dominated by and activated and highly immunosuppressive phenotype. Unlike blood, however, we did observe significant difference in sputum neutrophils in the airways, including elevated expression of CD177 and LOX-1 and reduced expression of the antimicrobial protein cathelicidin and the immunosuppression molecule arginase 1.

## Materials and methods

### Cohort description

Patients were recruited at the clinic after presenting with symptoms of TB and were tested using GeneXpert and confirmed by liquid culture. All patients gave written informed consent for a blood draw and induced sputum collection. The study protocol, data collection tools and associated consent forms were approved by the University of KwaZulu-Natal biomedical research Ethics Committee (BE285/16). The study protocol for blood collection from healthy donors and patients with active TB was also approved (BE022/13).

### Sputum and peripheral blood collection

Sputum samples were induced using PBS under supervision and samples were transported on ice to the lab and processing within one hour in a Biosafety level 3 facility. Blood samples were collected into EDTA tubes and processed within four hours of collection.

### NDN and LDN isolation using a Percoll gradient

To isolate neutrophils from blood, 6 ml of whole blood was mixed with 4 ml 5% dextran in 0.9% NaCl and incubated at RT for 30 mins. The layer containing neutrophils was then harvested and washed twice with HBSS without Ca^2+^/Mg^2+^ at 250 xg for 5 mins at RT. Isolated neutrophils were then separated into NDNs and LDNs using a Percoll density gradient. Briefly, Percoll (PureGene, Zeiningen, Switzerland) was made at concentrations of 100%, 81% 70% and 55% respectively. The isolated neutrophil pellet was resuspended in 55% percoll which was overlayed onto a 70% Percoll layer which itself was overlayed onto an 81% Percoll layer. This was then centrifuged at 720xg without brake at RT for 30 mins. NDNs were siphoned off from the 70%/55% boundary, with LDNs being siphoned off from the 81%/70% boundary. Cells where then washed once with PBS at 300xg for 10 mins at RT, and stained using antibody panels and analyzed by flow cytometry.

### Sputum processing

Induced sputum was mixed 1:2 (v/v) in 0.1% dithiothreitol (DTT, ThermoFisher Scientific) and incubated for 15 mins at 37 °C under agitation. Sputum/DTT mixture was mixed and passed through a 40 µm cell strainer. Cells were counted using an automated TC20 hemocytometer. Cells where then washed twice with PBS (300xg for 10 mins at RT), and stained using antibody panels and analyzed by flow cytometry.

### Flow cytometry analysis

Between 1 – 5 million cells were used for each flow cytometry panel. Cells were pelleted (300xg for 10 mins at RT) and stained with antibody panels with FC blocking (**Table 1**) for 20 mins at RT in the dark. Stained cells were subsequently washed twice with PBS and subsequently resuspended in 200 µl 2% PFA in PBS. Samples were then analyzed using a BD FACS Aria Fusion III, with all data being analyzed using FlowJo v10.8.1. For dimensionality reduction (t-SNE) analysis, individual donor fcs files were used to generate expression matrices containing measured intensities for each antibody in each of the two antibody panels. A total of 500 000 cells were used to create a t-SNE map in all cases, with an equal number of cells from each donor. Cell populations were then sub-setted and relative frequencies tested non-parametric analysis.

### Analysis of neutrophil protein levels in sputum and plasma

Neutrophil associated proteins were quantified by Enzyme-linked immunosorbent assays (ELISA) for both sputum and plasma. Sputum was processed as above, and then prepared for ELISA as per respective manufacturers instructions. Plasma was obtained from blood samples by centrifugation at 930xg for five mins and 1 ml plasma fractions were aliquoted and stored at -80°C. ELISAs for NGAL (HK330), MPO (HK324), Arginase-1 (HK386) and LL-37 (HK321) were purchased from Hycult Biotech (Hycult Biotech, Uden, Netherlands) while the TNFα quantikine kit was purchased from R&D systems (Bio-Techne, Minneapolis, United States). All ELISAs were run as per manufacturer’s instructions alongside standard protein curves.

### Statistical analysis

All statistical analysis were carried out in Graphpad using one way ANOVA to compare groups with Dunnetts correction for multiple comparison.

## Results

### Pulmonary TB is associated with neutrophilia and increased frequency of low-density neutrophils

**Figure 1:**
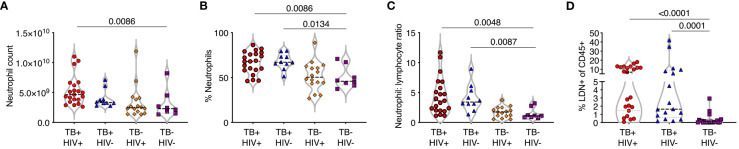
TB is associated with an expansion of normal and low-density neutrophils. **(A–C)** Whole blood counts from study participants with TB with and without HIV co-infection (TB+HIV+, TB+HIV-) with HIV alone (TB-HIV+) and controls with neither HIV or TB-HIV), showing **(A)** neutrophil count per ml whole blood, **(B)** percentage of nucleated cells **(C)** neutrophil:lymphocyte ratio. **(D)** Low density neutrophils (LDN) purified by Percol gradient and shown as a percentage of total CD45% cells. Only significant differences shown.

Full blood count data was obtained from participants with active TB disease (ATB), with and without HIV infection, attending TB clinics and sampled on the day of diagnosis. Control samples were obtained from non-TB infected volunteers without any signs or symptoms of TB disease. Neutrophil frequency and percentage were significantly higher in ATB regardless of HIV status compared to controls (**Figures 1A, B**). In addition, the neutrophil-lymphocyte ratio (NLR), a marker of systemic inflammation for several diseases (26, 27), was significantly elevated compared to controls in both ATB diseased groups (**Figure 1C**). Neutrophils are typically high-density cells that separated out with red blood cells during standard PBMC isolation procedures. However, a fraction of neutrophils separates out with the PBMC layer, due to a lower cell density. These low-density neutrophils are considered proinflammatory and are associated with several human diseases including sepsis. The frequency of low-density neutrophils (LDN) present was determined by centrifugation and was also found to be highly elevated in ATB regardless of HIV (**Figure 1D**). These data suggest a strong blood neutrophil response to ATB, consistent with other studies (28, 29).

### Phenotyping of normal-density and low-density neutrophils using flow cytometry

**Figure 2:**
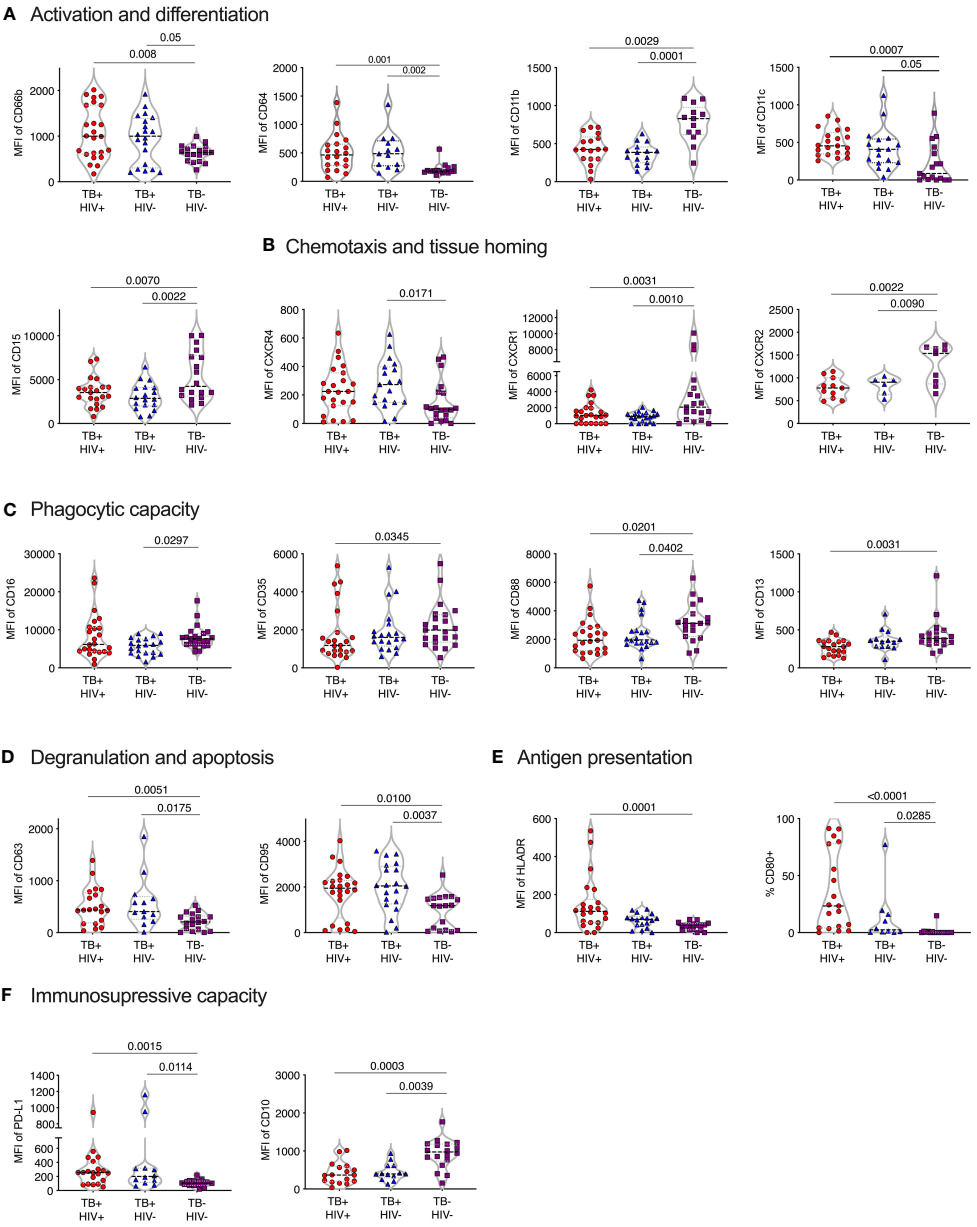
Phenotype of normal density blood neutrophils (NDN) highly skewed in active TB with and without HIV infection. Mean fluoresce intensity (MFI) for neutrophil surface markers grouped by functional category **(A–F)**. Only markers with significant differences between groups shown, with p-values indicated by Kruskal-Wallis test.

Normal-density (NDN) and low-density neutrophils (LDN) were separated by centrifugation and stained with two antibody panels encompassing known phenotypic and functional neutrophil markers (**Table 1**). Except for CD177, these markers did not typically differentiate positive and negative populations but rather varied in expression levels. Therefore, differences in expression levels of these markers are displayed as Median Fluorescent Intensity (MFI) level. Markers displaying significant differences in MFI are shown in **Figure 2**, in which markers have been categorized according to function. It is important to note, however, that these categories are not always distinct, and several surface molecules are known to have overlapping functions.

**Table 1:** Full list of antibodies used in the phenotyping of neutrophils through flow cytometry.

Marker	Clone	Fluorophore	Manufacturer	Cat number	Panel
CD66b	G10F5	BV421	BD	BD562940	1
HLA-DR	G46–6	BV605	BD	BD562845	1
CD11b	ICRF44	BV510	BD	BD563088	1
CD11c	B-ly6	BV650	BD	BD563404	1
CD14	M5E2	BV711	Biolegend	301838	1
CD181	8F1	PE-Cy5	Biolegend	320610	1
CD184	12G5	PE-Cy7	Biolegend	306514	1
CD177	MEM-166	PE	BD	BD564239	1
CD88	S5/1	APC	Biolegend	344310	1
CD35	E11	BUV395	BD	BD565328	1
CD16	3G8	BUV496	BD	BD564653	1
CD69	FN50	BV785	Biolegend	310932	1
CD62L	DREG-56	PerCP/Cy5.5	Biolegend	304824	1
CD66b	G10F5	BV421	BD	BD562940	2
CD14	M5E2	BV711	Biolegend	301838	2
CD64	10.1	BV510	Biolegend	305028	2
CD63	H5C6	PECy7	Biolegend	353010	2
CD274	29E.2A3	BV605	Biolegend	329724	2
CD33	P67.6	APC	BD	BD345800	2
CD80	L307.4	BV650	BD	BD564158	2
Lox-1	15C4	PE	Biolegend	358604	2
CD182	5E8	PerCP/Cy5.5	Biolegend	320718	
CD15	W6D3	BV510	Biolegend	323028	
CD56	HCD56	APC-Cy7	Biolegend	318332	
CD45	HI30	AF700	Biolegend	304024	
CD35	AKT3	APC-Cy7	Biolegend	317342	
CD206	19.2	PE-Cy5	BD	BD551136	
CD31	WM59	BV605	Biolegend	303122	
CD32	FLI8.26	APC	BD	BD559769	
CD197	G043H7	PerCP/Cy5.5	Biolegend	353220	
CD38	HIT2	PE-Cy7	Biolegend	303516	
CD1c	L161	PE-Cy7	Biolegend	331516	
CD123	7G3	PE-CF594	BD	BD562391	
CD86	2331	PE-Cy7	BD	BD561128	
CD163	GHI/61	FITC	BD	BD563697	
CD83	HB15e	PE-Cy5	Biolegend	305310	

#### Activation and differentiation

In general, NDN displayed an activated phenotype in ATB, compared to controls, with significantly elevated expression of surface CD66b and CD64 (30–33) (**Figure 2A**). In addition, CD11c was upregulated in ATB, which, although typically associated with dendritic cells, is expressed on activated neutrophils and, together with CD64, is an effective biomarker of sepsis in humans (34). However, the expression of CD11b (CR3), which is typically upregulated following neutrophil activation and during bacterial infection (35) was significantly lower in ATB. In addition, CD15 was significantly lower, potentially indicating the presence of more immature neutrophils.

#### Chemotaxis and homing markers

The expression of CXCR4, which is central for neutrophil retention in bone marrow via the SDF-1 gradient (36, 37), was significantly upregulated in ATB without HIV compared to controls (**Figure 2B**). In contrast, and again consistent with an activated phenotype (38), the expression of the two IL-8 receptors, CXCR1 and CXCR2, was downregulated in ATB. The relative upregulation of CXCR4 and reduction of CXCR2 is also consistent with the return of aging neutrophils back to the bone marrow (39).

#### Phagocytic molecules

The expression of CD35 (CR1), CD88 (C5aR), CD16 (FcRIII) and CD13 phagocytic receptors were all reduced in ATB, although this did not reach significance in all groups (**Figure 2C**). Down regulation of CD16 has been associated with reduced functional capacity and increased apoptosis (40), while CD35 and CD88 expression is reduced in chronic inflammation and correlates strongly with impaired phagocytic capacity (41–43). In addition, blocking of CD16 and CD35 has been shown to specifically reduce phagocytosis of *Mtb* by neutrophils (44). Together these data are consistent with a reduction of neutrophil phagocytic capacity in ATB.

#### Degranulation and apoptosis

The expression of the azurophillic granule protein CD63 on the surface of neutrophils, which is a marker of neutrophil activation and degranulation, was increased in ATB. CD95 expression was also significantly increased in ATB patients compared to controls, consistent with a pro-apoptotic phenotype (**Figure 2D**).

#### Expression of co-stimulatory and immunosuppressive receptors

Expression of the T-cell immunosuppressive molecule CD274 (PDL-1) was increased ATB, regardless of HIV status. However, CD10 expression was significantly reduced **Figure 2F**), which is indicative of immature neutrophils that activate T-cells (45, 46). Potentially in line with this, both HLA-DR and the co-stimulatory molecule CD80 were elevated in TB disease, consistent with an upregulation of antigen presentation activity (**Figure 2E**) (47, 48).

No significant differences in expression of the phenotypic markers measured were observed between ATB participants with and without HIV co-infection. Together these data indicate a profound change in neutrophil phenotype in TB disease, largely consistent with activation, degranulation and apoptosis, in addition to reduced phagocytic function, and increased cross talk with T-cells.

### Comparison of NDN and LDN phenotypes in peripheral blood

Next, we examined the phenotype of LDN from the same subjects. Like NDNs, LDNs from ATB had an increased expression of HLA-DR, CXCR4, CD95, PDL-1 and CD80, and decreased expression of CD15, generally to a greater extent, but other phenotypic changes, such as for CXCR1 and 2 were not observed (**Supplementary Figure S2**). In addition, CD177, which is typically expressed by activated neutrophils (49) and was not differentially expressed on NDN in ATB, was highly upregulated on LDN in ATB. Likewise, LOX-1, which has been linked to immunosuppressive neutrophils and is upregulated during sepsis (50) was unchanged on NDN, but was highly upregulated on LDN in ATB. Together these data also suggest that the phenotype of activated and immunosuppressive neutrophils observed in NDN is largely reflected in LDN. However, the fact that key markers such as CD177 and LOX-1 were differentially expressed caused us to examine the general pattern of all neutrophil markers studied in NDN and LDN.

#### Comparison of NDNs and LDNs from controls

The expression level of opsonophagocytic receptors (CD88 and CD35), chemotaxis and tissue homing receptors (CXCR2, CD62L and CD177), antigen presentation markers (HLADR, CD11c) as well as the maturity and immunoregulatory receptor, CD10, were all significantly lower in LDNs compared to NDNs in healthy controls (**Supplementary Figure S2**). In contrast, CD66b, was higher in LDN, as was CD16 and CD15, in line with previous reports (20). Expression of CD63 was also higher in LDNs, supporting the hypothesis that LDNs represent or contain neutrophils that have degranulated. The other phenotypic markers measured were not significantly different between these two neutrophil subsets. Overall, these differences suggest that, under steady state conditions, LDNs are more degranulated and less functional than NDNs. However, the patterns of markers associated with other neutrophil states, such as activation, in both NDN and LDN were somewhat conflicting.

### Distinct clusters of neutrophil subsets identified in NDN and LDN

**Figure 3:**
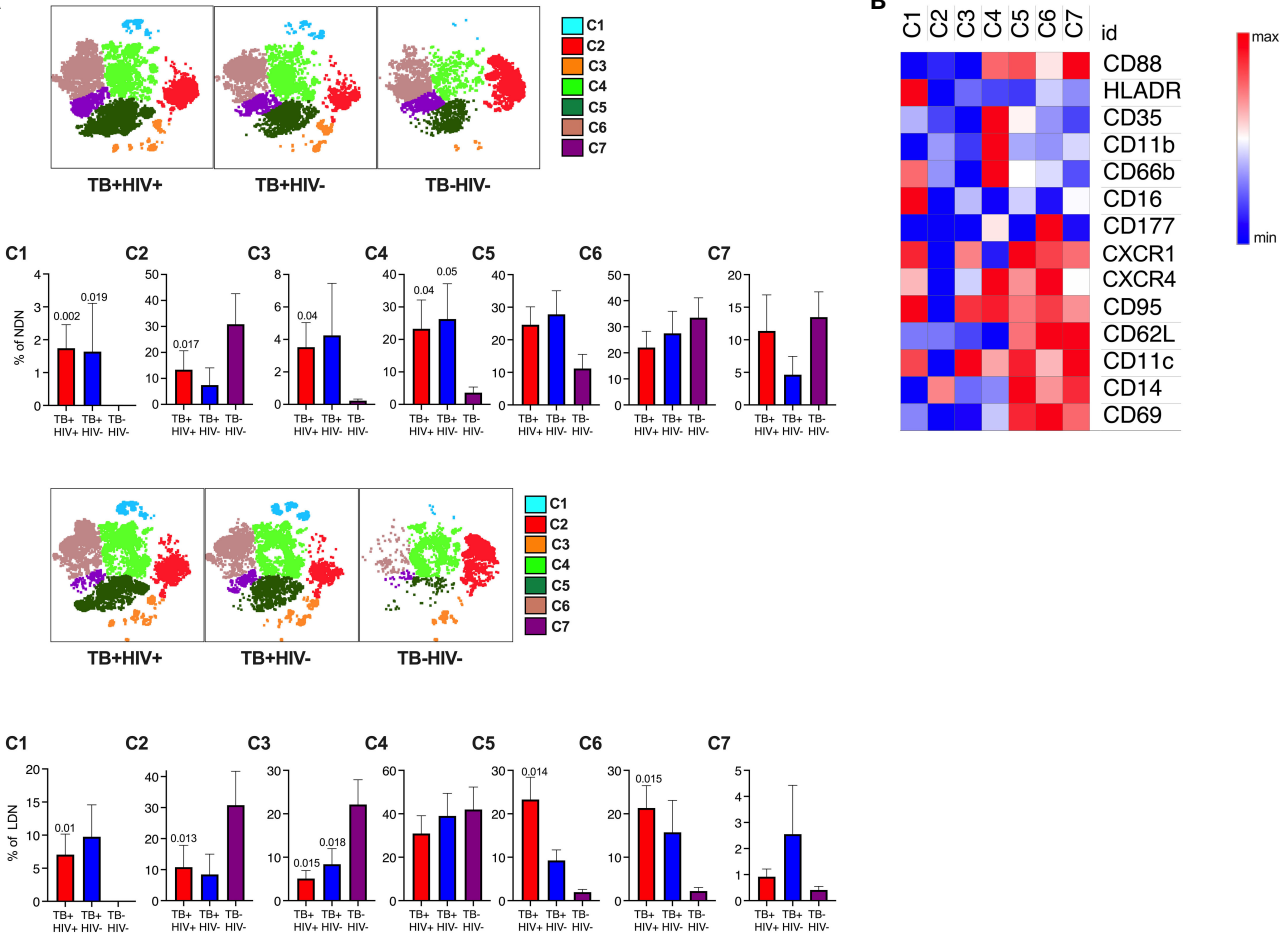
Subsets of NDN and LDN identified by tNSE analysis using FlowJo. Neutrophil subsets/phenotypes identified by clustering using unbiased dimensionality reduction tNSE analysis for NDN **(A)** and LDN **(D)**. The heat map defining average expression value by MFI for marker is shown in panel **(B)**. The mean frequency and SEM of each cluster shown for each group for NDN **(C)** and LDN **(E)**, with p-value indicating significant differences to TB-HIV- controls by Kruskal-Wallis test.

Having observed complex and potentially conflicting neutrophil phenotypes, we next used an unsupervised t-SNE analysis of NDN and LDN to determine if these related to distinct neutrophil subsets. This approach identified 7 clusters for each antibody panel (**Figures 3A, B, D**; **Supplementary Figures S3A, B, D**). In **Figure 3**, cluster 1 forms a distinct subset distinguished by high expression of HLA-DR and CD66b and low expression of CD35 and CD88, consistent with a highly activated and functionally impaired subset. In line with the above observations, this subset was significantly expanded in both NDNs and LDNs in ATB (**Figures 3C, E**) (51). Cluster 4 was also highly expanded in ATB on NDN, and displayed elevated expression of CD11b, canonically associated with neutrophil activation and potentially explaining the unexpected overall reduction of CD11b. This cluster also displayed a reverse migratory phenotype of low CXCR1 and high CXCR4, potentially indicating neutrophils that have migrated out of the inflamed lung (52, 53). Interestingly, these cells retained expression of CD35 and CD88, which, like CD11b were downregulated overall. Again, consistent with the above data, cluster 6, which was significantly upregulated in LDN alone, was defined by unique expression of CD177. Cluster 2, 6 and 7 were the dominant subpopulations observed in NDN of uninfected controls, implying these represent steady state neutrophils subsets, of which cluster 2 was significantly down regulated in ATB. Similarly, cluster 2, 3 and 4 were by far the dominant subsets in healthy LDN, of which 2 and 3 were significantly reduced in ATB.

In the second panel, the immunosuppressive phenotype is apparent (**Figures S3C, E**). Clusters 1 and 4 expressed high levels of PDL-1 and were both highly elevated in NDN and LDN from participants with ATB. In addition, cluster 1 expressed highly levels of CD10, consistent with a T-cell suppressive phenotype (46, 54), while the clusters lacking CD10 (3 and 7, consistent with a T-cell stimulatory phenotype) also lack PDL-1 expression. Furthermore, clusters 1 and 4 uniquely express CD80 as observed for tumor associated immunosuppressive neutrophils (55). Clusters 2 and 3 expressed elevated LOX-1, which has been linked with immunosuppressive activity, although this does not reach statistical significance. Interestingly, these populations express elevated CD137, which is linked to a protective response to Gram-positive but not Gram-negative bacteria (56). By far the dominant NDN cluster in controls is cluster 5, which is significantly reduced in ATB. In LDN this cluster is also dominant in controls, together with cluster 6, which uniquely expresses CD63, consistent with hypothesis that a proportion of LDN is made of degranulated neutrophils. Interestingly, however, this population is significantly reduced in LDN from ATB but expanded in NDNs.

For both panels, no significant differences were observed between HIV infected and uninfected participants. This unbiased sub clustering approach indicates diverse sub-populations or neutrophil states in blood, which are highly skewed by ATB. Overall, it confirms the association with highly activated and potentially dysfunctional neutrophils together with the expansion of separate populations of immunosuppressive neutrophils.

### Neutrophil phenotypes in the lungs

**Figure 4:**
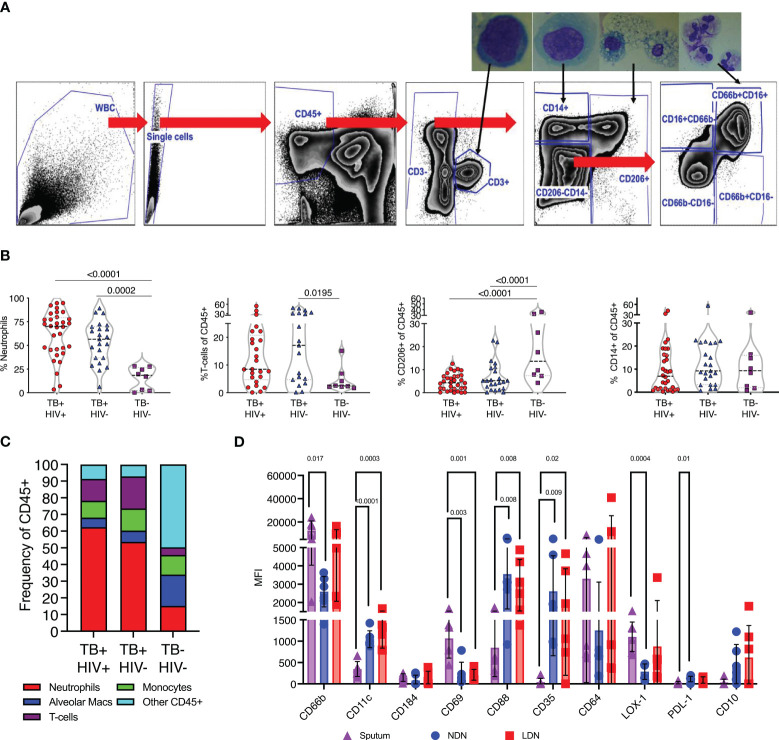
TB associated with high neutrophil abundance in sputum. **(A)** Flow gating strategy for identifying cell sub-sets within fresh sputum samples. Each of the main subsets was sorted using a FACS ARIA cell sorter and sorted cells spun on to microscope slides using a cytospin for visual confirmation of appropriate morphology, as shown. **(B)** The relative abundance of CD66b+ neutrophils, CD3+ T-cells, CD206+ alveolar macrophages and CD14+ monocytes. Significant differences indicated by p-values, by Kruskal-Wallis test. **(C)**. A summary of the average cellular make of sputum samples from TB+HIV-, TB+HIV+ and TB and HIV uninfected controls. **(D)** Phenotypic characteristics of sputum neutrophils compared to NDN and LDN from matched blood samples from Non-TB Non-HIV infected controls, significant difference shown by p-value (Kruskal-Wallis).

Having observed profound differences in blood neutrophils in TB disease, we next determined if these changes were also reflected at the site of disease in the lung airways. To do this we studied the cellular content of sputum samples collected from participants attending the TB clinics at the time of diagnosis and before the initiation of TB treatment. Study participants had microbiologically confirmed TB by GeneXpert and liquid culture. In addition, samples were obtained from symptomatic TB negative participants who received a negative gene-expert result at the clinic and who were subsequently found to be culture negative. Sputum samples were collected into buffered media and transported to the lab within 1 hour of collection and incubated with DTT to break up mucins and liberate cells present. Subsequent flow cytometric analysis was then used to quantify cellular contents according to the gating strategy shown in **Figure 4A**. To confirm the cellular identity, CD3+ T-cells, CD14+ myeloid cells, CD206+ alveolar macrophages and CD66b+CD16+ neutrophils were sorted separately using a FACS ARIA contained with the BSL-3. Sorted populations were spun onto microscope slides and visualized, revealing the canonical morphologies associated with each cell type (**Figure 4A**).

#### Neutrophils accumulate in sputum during active TB

Quantification of sputum cellular contents based on these gates revealed a profound enrichment of neutrophils in ATB compared to controls, irrespective of HIV coinfection (**Figure 4B**). CD3+ve T-cells were also enriched compared to controls, consistent with a recruitment of these cells to TB diseased lung tissue, although this only reached significance for HIV co-infected participants (57). In contrast, the proportion made up by alveolar macrophages was significantly reduced in ATB, although maybe due to result from the enrichment of the neutrophils and T-cells rather than a loss of this important airway resident subset. From this data neutrophils were found to make up 50–60% of the cellular content of sputum of individuals with ATB, irrespective of HIV, but only 10% in respiratory symptomatic TB negative controls (**Figure 4C**).

First, we examined the phenotype of sputum neutrophils from TB negative controls compared to NDN and LDN from matched blood samples. It is important to note the non-TB controls in this case were symptomatic, and these neutrophil phenotypes may not represent a non-disease situation. None-the-less, this analysis clearly indicates differential neutrophil phenotypes in blood and sputum. Notably this includes increased expression of CD69, which is expressed on activated neutrophils (58), and higher expression of CD66b than NDN (**Figure 4D**). Sputum neutrophils expressed significantly lower levels of CD11c, and the phagocytic receptors CD35 and CD88, potentially suggested reduced functionality. They expressed higher levels of LOX-1 than blood NDN, consistent with an immunoregulatory role, but less PDL-1 and were negative for CD10. Markers not shown were not significantly different.

### Sputum neutrophils have a distinct phenotype in TB infected individuals

**Figure 5:**
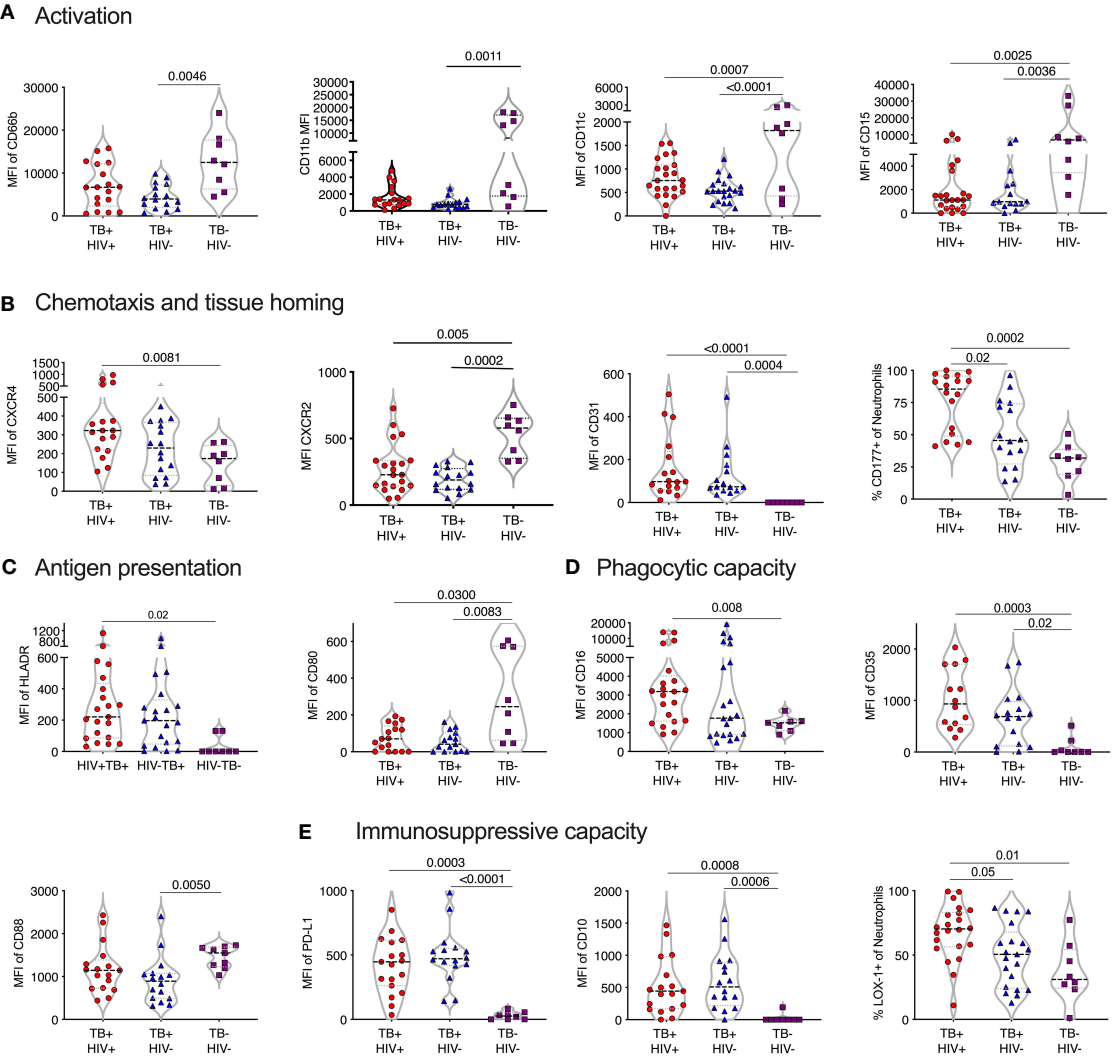
TB skewed phenotype of sputum. Mean fluoresce intensity (MFI) for neutrophil surface markers grouped by functional category **(A–E)**, LDN and NDN were not distinguished in sputum. Only the markers with significant differences between groups are shown, with p-values indicated by Kruskal-Wallis test.

Comparison of sputum neutrophils present in non-TB controls with those isolated from ATB revealed consistent and striking differences (**Figure 5**). In contrast to blood neutrophils during TB disease, sputum neutrophils expressed lower levels of CD66b compared to non-TB controls. Together with the reduced CD15 expression levels observed, this is consistent with a proapoptotic phenotype (59), although CD95 expression levels were not different. In addition, and similar to blood, sputum neutrophils from ATB expressed elevated levels of CXCR4, which is known to upregulate on aged neutrophils and is associated with migration back to the bone marrow (60). Again, as in blood, CXCR2 was significantly down regulated in ATB in sputum. Unlike blood, however, the frequency of CD177+ neutrophils was significantly elevated, as was the expression of CD31, which both promote neutrophil transmigration (61). Together these data suggest an accumulation of ageing and pro-apoptotic neutrophils in TB infected lungs with the potential to transmigrate out of the lung and back into the blood. Similar to blood, sputum neutrophils from TB infected participants expressed significantly lower levels of CD88, which impairs phagocytic capacity in general (42). However, CD35 expression was elevated compared to non-TB controls and CD16 was no longer reduced, as in the blood (**Figure 5**). As mentioned, together these molecules are crucial for phagocytosis of opsonized *M.tb* (44), suggesting that this capacity may be retained by sputum neutrophils during active disease.

Strikingly, in TB-negative individuals, sputum neutrophils expressed lower levels of PDL-1 than in blood. However, in ATB this was highly elevated, consistent with an immunosuppressive phenotype. Sputum neutrophils from ATB also expressed significantly higher levels of CD10 and LOX-1 (**Figure 5**). Furthermore, the expression levels of PDL-1, CD10 and LOX-1 was higher than in match blood samples from TB diseased participants (**Supplementary Figure 4**). This immunosuppressive phenotype was further supported by the increased expression of the antigen presenting molecule HLA-DR, but decreased expression of the co-stimulatory receptor CD80, which was elevated in the blood. Therefore, sputum neutrophils in infected individuals express a consistent T-cell immunosuppressive phenotype. Overall, as in blood, neutrophil phenotype was similar in HIV infected and uninfected participants, although LOX-1+ and CD177+ neutrophils were significantly more frequent in individuals with HIV co-infection, suggesting a potential greater impact of HIV in the lung compartment.

### Neutrophil associated protein levels in blood and sputum during ATB

**Figure 6:**
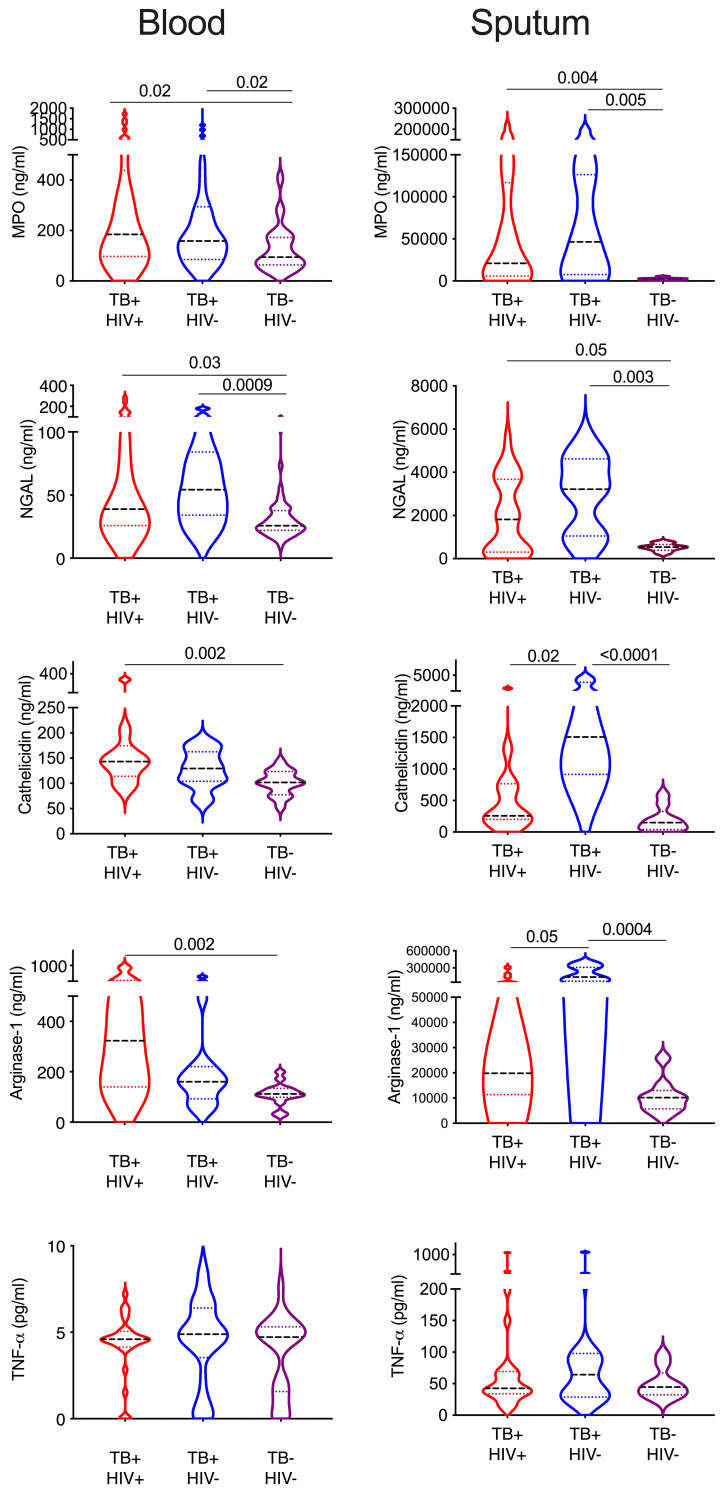
Sputum from HIV co-infected individual contains lower neutrophils proteins. Key neutrophil proteins were measured in matched blood plasma and decellularized sputum using commercial ELSIA assays. All significant differences are shown by p-value (Kruskal-Wallis test).

Finally, we sought to confirm the presence of enriched neutrophils in the sputum of TB infected subjects by measuring neutrophil related proteins via ELISA (**Figure 6**). The same proteins were measured in matched plasma samples to compare the blood and lung compartments. Myeloperoxidase (MPO) is an important antimicrobial protein that is abundant in neutrophils and often used as a marker of this cell type, although it can also be found in myeloid and lymphoid cells (62). MPO was significantly increased in both plasma and sputum from TB participants irrespective of HIV, and this was especially pronounced in sputum and highest in HIV uninfected subjects (7.8x higher TB+HIV+, and 17x higher in TB+HIV- compared to TB-HIV-). Likewise, neutrophil gelatinase-associated lipocalin (NGAL), an antimicrobial peptide that limits iron availability and is highly expressed by neutrophils during inflammation, was elevated in both plasma and sputum in TB infected participants; but to a much greater extent in sputum (5.6x higher and 7x higher in TB+ HIV+ and TB+HIV-, respectively, compared to TB-HIV-). For cathelicidin (LL37), an antimicrobial protein which has been shown to be important in the killing of *Mtb*, levels were significantly elevated in plasma for TB+HIV+ participants only. In sputum, however, cathelicidin levels were only highly elevated in TB infected subjects without HIV (10x higher than TB-HIV-) and were significantly higher than TB infected subjects with HIV (5.9x higher than TB+HIV+). A similar trend was observed for arginase 1, an enzyme that enhances Mtb killing and limits pathogenic T-cell activation. As with cathelicidin, arginase 1 is significantly elevated in only the blood of TB+HIV+ participants, but in the sputum, it is only elevated in TB+HIV- participants compared to TB-HIV- (13.9x higher) and TB+HIV+ study subjects (7.2 x higher). No significant differences in TNF-a levels were seen in either blood or sputum of TB and non-TB controls. Overall, this data confirms the highly significant enrichment of neutrophil proteins in the sputum of TB infected subjects. Furthermore, our findings suggest reduced expression of important antimicrobial peptides in lungs of HIV co-infected individuals that are not reflected in the blood.

## Discussion

Neutrophils play a complex role in the immunopathogenesis of TB that remains poorly understood, exacerbated by the growing appreciation that neutrophils exist in multiple distinct functional states. In this study we investigate the frequency and heterogeneity of neutrophils in blood and in the lung airways by sampling sputum. ATB was associated with an enrichment of neutrophils in both compartments, particularly in sputum, where they were by far the dominant cell type. In normal density neutrophils (NDN) from blood, ATB was associated with an activated and proapoptotic phenotype expressing changes consistent with reduced phagocytic capacity and increased immunoregulatory function. Low density neutrophils (LDN) were also significantly increased in frequency in ATB. This subset is generally considered to be proinflammatory and has reduced phagocytic capacity which was supported by comparison between NDN and LDN in controls. In addition, ATB was associated with further phenotypic changes in LDN consistent with those seen in NDN.

Using high dimensional clustering to visualize distinct neutrophil subsets confirmed these observations, showing significant upregulation of clusters with activated and functionally impaired phenotypes in ATB in both NDN and LDN. In addition, this analysis supports the enrichment of distinct immunosuppressive neutrophil subsets in ATB, some of which express high level of PDL-1 together with the T-cell co-stimulatory molecule CD80, and others which lack PDL-1 but express LOX-1. Expression of CD80 and HLA-DR on neutrophils is associated with the acquisition of antigen presenting functions on neutrophils (47), and, although not on the same panel, ATB was also associated with distinct HLA-DR high neutrophil subset. The expression of PDL-1, HLA-DR and CD80 is observed on tumor associated neutrophils and associated with suppression of T-cell activity. In addition, one subset of PDL-1 high neutrophils also expressed high levels of CD10, which has been shown to identify neutrophils that suppress T-cell activation (46). This suggests the active TB leads to the expansion of neutrophil subsets that directly inhibit Mtb specific T-cells via both the PDL-1 axis and the interaction between CD80 and CTLA4. Importantly, PDL-1 expression, whilst low on sputum neutrophils from TB-uninfected subjects was highly elevated in ATB, as was CD10, implying this T-cell suppressive phenotype is likely to be higher in the lung. M.tb infected mice lacking PD-1 had increased neutrophil recruitment and severe lung pathology which led to their early death compared to wild type mice (63), suggesting that PDL-1 upregulation is a negative feedback loop that limits excessive neutrophil recruitment. On the other hand, elevated PD-L1 expression may be counterproductive, as it could suppress the protective Mtb specific T-cell responses during infection (64, 65). In addition, PDL-1 expression by neutrophils has recently been shown to prolong neutrophil survival in patients with sepsis via activation of AKT dependent survival signaling (66). In conditional knockout mice, removal of PDL-1 improved survival by reducing neutrophil recruitment and subsequent tissue damage. Similarly, again in mice, PDL-1 expression was recently shown to promote neutrophil retention in lung tissue and enhance susceptibility to bacterial infection, which could be reversed by anti-PDL-1 treatment (67). PDL-1 expression on neutrophils was also found to prevent autophagy and thus exacerbate endotoxin induced lung injury by prolonging NETosis (68), a mechanism also thought to be important in TB pathology (69). Although LOX-1 has been associated with T-cell suppression by neutrophils, particularly in cancer (70), like PDL-1, LOX-1 is upregulated on neutrophils during sepsis, and deletion of LOX-1 reduced neutrophil driven lung pathology (50). Indeed, both LOX-1 and PDL-1 expressing neutrophils subsets were highly upregulated in blood and lung of patients with severe COVID (71). Thus, these neutrophil subsets may play dual roles in ATB, suppressing T-cell activity, whilst exacerbating lung immunopathology. For PDL-1 in particular, blockade of this molecule in experimental setting has reduced neutrophil associated lung injury associated with bacterial infection. The use of anti-PD1 blockade in cancer therapy has led to numerous cases of TB reactivation, indicating this pathway may be important in maintain latency (72). However, given the potential importance of PDL-1 in promoting neutrophil immunopathology in TB disease, blockade of the pathway might prove beneficial in conjunction with conventional anti-TB drugs.

Another striking observation from the in-depth phenotyping of blood and airway neutrophils comes from the expression of molecules associated with migration. In both blood and sputum, ATB is associated with significant upregulation of CXCR4 and downregulation of CXCR1 and CXCR2. The latter are receptors for IL-8 and promote release of neutrophils from bone and recruitment to site of inflammation. CXCR2 in particular has been shown to be essential from recruitment of neutrophils to the lung (10). Down regulation of both receptors has been associated with neutrophil activation in numerous disease settings including COPD (73). Likewise, CXCR4 is upregulated in chronic inflammatory lung disease and is associated with recruitment of apoptotic neutrophils from the lung back to the bone marrow (74). Consistent, with this, high dimensional analysis of blood neutrophils revealed a distinct population of CXCR4hi CXCR1lo neutrophils that also expressed CD11b, CD66b and CD95, consistent with highly activated, proapoptotic neutrophils that may be recruited back to the bone marrow. In the lung airways, the same down regulation of CXCR1 and upregulation of CXCR4 was observed. In addition, this is accompanied by upregulation of CD31 (PECAM-1), which is important for neutrophil migration into the airways via homophilic binding of CD31 (75). Interestingly, CD177 was also upregulated on sputum neutrophils in ATB, which is known promote transmigration also via binding to CD31 (61). This data suggests that CD31 may play an important role in the recruitment of neutrophils to the lung alveolar space during active TB. Like alveolar macrophages, neutrophils can reverse migrate from airways back into tissue during infection (76). Reverse migration is thought to be important event in several settings, including sepsis, where it may help to resolve inflammation or promote tissue damage (77). Given that sputum neutrophils are known to be heavily infected with mTB during ATB (78), it is possible that neutrophils play a role on disseminating Mtb within lung tissue and systemically. Indeed, as CXCR4hi CXCR1lo neutrophils are recruited back to the bone marrow, it is also possible that this is a mechanism by which Mtb is translocated to bone marrow, where it can be found even following TB cure (79).

The functionality of neutrophils in blood and sputum was not directly assessed in this study. However, blood neutrophils expressed significantly lower levels of the phagocytic markers CD16, CD35, CD88 and CD13. As mentioned, blockade of CD16 and CD35 has been shown to reduce phagocytosis of Mtb (44). In that study blockade of CD88 had no effect, however, in critically ill patients with suspected pneumonia, only surface CD88 expression was found to correlated with reduced phagocytic capacity of blood neutrophils (42). Similarly, cross linking of CD13 has recently been shown to induce phagocytosis on neutrophils (80). The FCyR CD64, in contrast, is highly upregulated in ATB, consistent with its potential useful role as a clinically useful marker of bacterial infection (81), including Mtb (82). However, despite facilitating phagocytosis, elevated CD64 in tuberculosis is associated with decreased phagocytic potential (83). This is potentially explained by our data showing a down regulation of most phagocytic receptors during ATB, consistent with an overall reduction in phagocytic capacity. Interestingly, down regulation of CD88 in the above study, compared to controls, was only observed in blood and not bronchioalveolar lavage fluid (42). Here, we did observe a significant down regulation of CD88 in sputum neutrophils from TB+HIV- participants compared to controls, but it was modest. In addition, CD16 and CD35 expression increased. This data suggests that airway neutrophils are likely to retain phagocytic activity, which is consistent with the observation that they are the most abundantly infected cell type in this compartment. However, whether they are able to kill phagocytosed Mtb is not clear and the data is conflicting (84).

Finally, we did not observe many phenotypic differences between ATB with and without HIV co-infection in blood. However, in sputum the expression of CD177 and LOX-1 was significantly elevated and two key neutrophil associated proteins, cathelicidin and arginase-1 were both significantly reduced in ATB co-infected with HIV. The frequency of neutrophils in these same sputum samples was not significantly different and, indeed tended to be higher in co-infected subjects. This suggests that HIV co-infection may lead to further functional impairment of neutrophils in the lung compartment, which might contribute to immunopathology in these individuals.

In conclusion, detailed phenotyping of blood and sputum neutrophils revealed profound skewing of this important immune subset in ATB in both HIV infected and uninfected individuals. The enrichment of highly activated and immunoregulatory subsets is broadly consistent between the blood and airways. Importantly, these phenotypic changes suggest diverse ways by which the skewing of neutrophils in the lung may exacerbate the immunopathology of TB. These data also suggest potential avenues for reducing neutrophil mediated lung pathology at the point of diagnosis.

## Data availability statement

The raw data supporting the conclusions of this article will be made available by the authors, without undue reservation.

## Ethics statement

The studies involving humans were approved by University of KwaZulu Natal Biomedical Research Ethics Committee (BREC). The studies were conducted in accordance with the local legislation and institutional requirements. The participants provided their written informed consent to participate in this study.

## Author contributions

SN: Formal analysis, Investigation, Methodology, Writing – original draft. MC: Data curation, Formal analysis, Investigation, Visualization, Writing – original draft. LN: Conceptualization, Writing – review & editing. FK: Project administration, Writing – review & editing. MM: Project administration, Writing – review & editing. ZM: Project administration, Writing – review & editing. LM: Project administration, Writing – review & editing. YM: Project administration, Writing – review & editing. SM: Formal analysis, Writing – review & editing. MH: Project administration, Writing – review & editing. AL: Conceptualization, Methodology, Supervision, Writing – original draft, Writing – review & editing.
