# Supplementary figures and images for: Detailed phenotyping reveals diverse and highly skewed neutrophil subsets in both the blood and airways during active tuberculosis infection
Source: Front Immunol. 2024 Jun 14;15:1422836. doi: 10.3389/fimmu.2024.1422836 (PMC11212598; doi:10.3389/fimmu.2024.1422836)

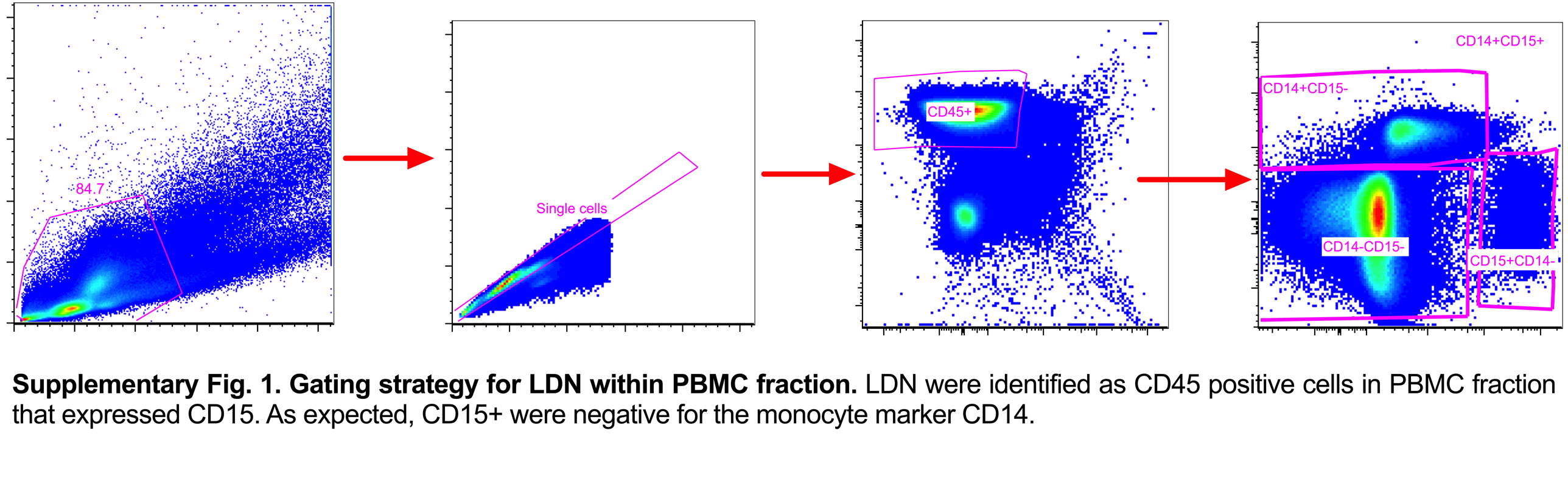

Supplement: Supplementary file 1 [file Image_1.jpeg]

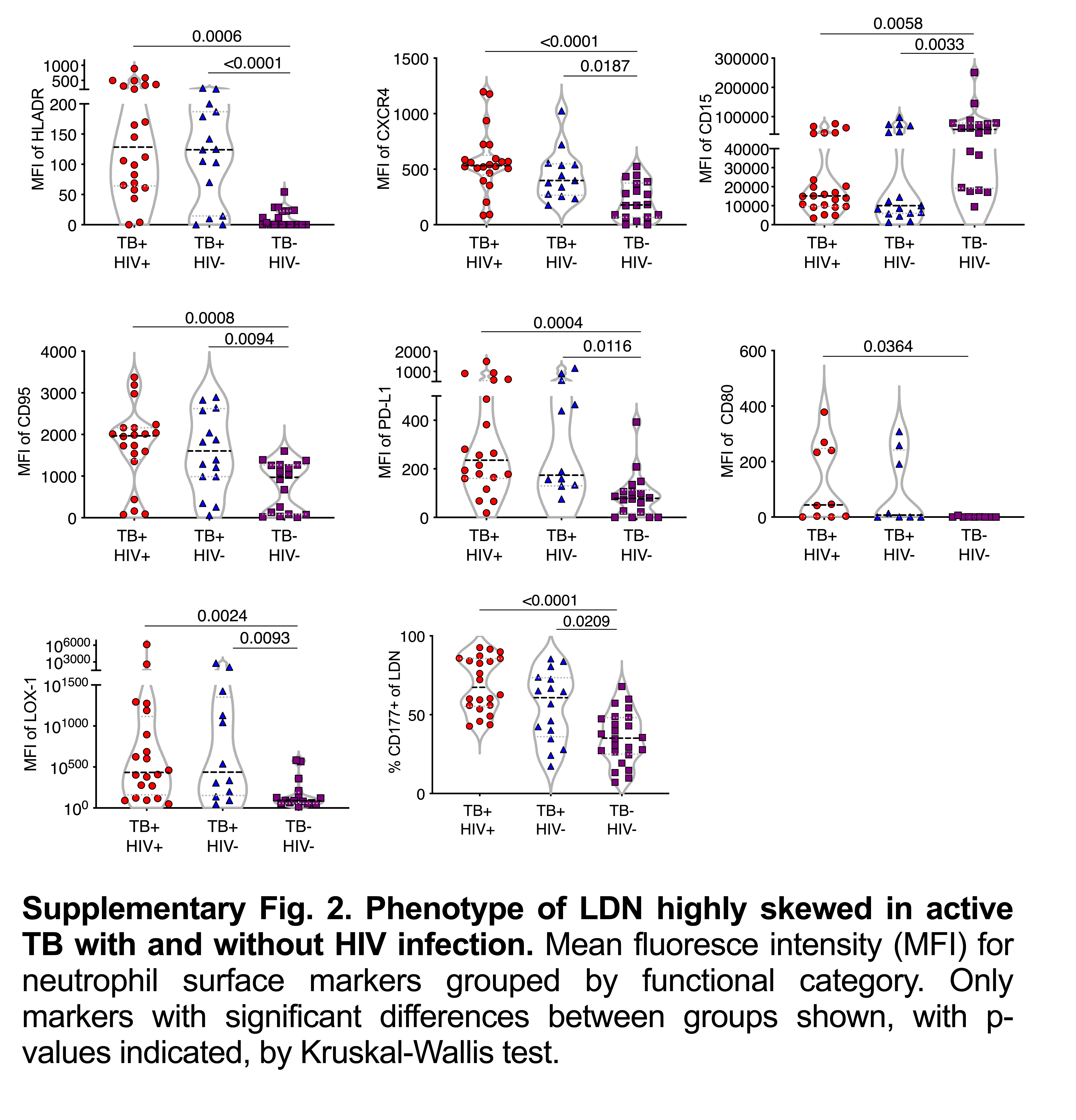

Supplement: Supplementary file 2 [file Image_2.jpeg]

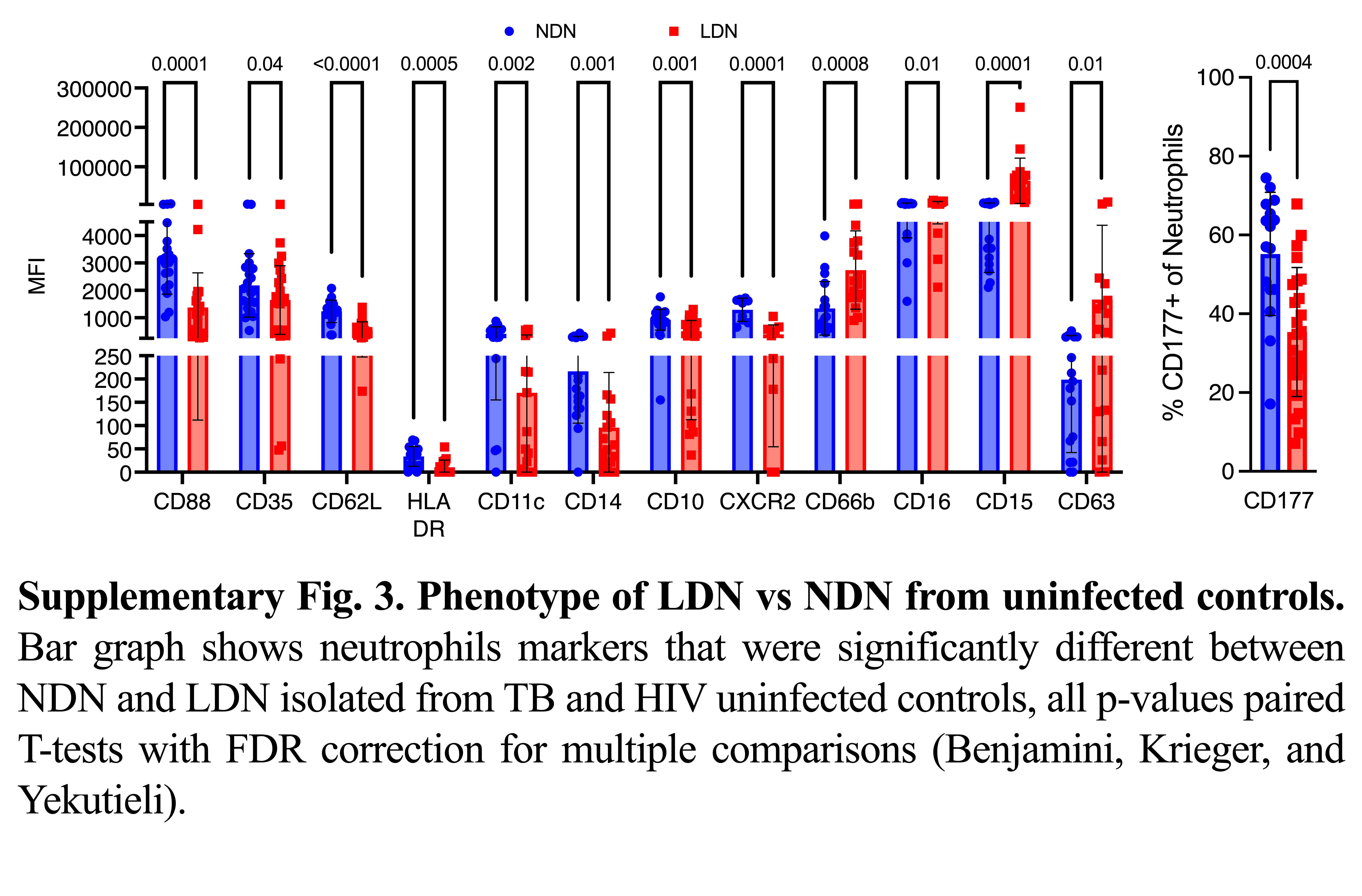

Supplement: Supplementary file 3 [file Image_3.jpeg]
